# Microbial diversity in leg ulcer and skin in Sickle Cell disease

**DOI:** 10.1016/j.htct.2026.108550

**Published:** 2026-10-16

**Authors:** Franciane Mendes-Oliveira, André Rolim Belisário, Marina Lobato Martins, Roberta Cristina Ruedas Martins, Lucas Augusto Moyses Franco, Nazareno Scaccia, Joyce Vanessa da Silva Fonseca, Isabel Cristina Gomes Moura, Valquíria Reis de Souza, Silvia Figueiredo Costa, Ian Valença, Eduarda Bolina-Santos, Fabíola Gomes Mendes, Elizabeth Castro Moreno, Alice Timponi, Jordana Grazziela Alves Coelho dos Reis, Olindo Assis Martins-Filho, Anna Bárbara Carneiro-Proietti, Ester Cerdeira Sabino

**Affiliations:** a Faculty of Medicine, University of São Paulo, São Paulo, SP, Brazil; b Hemominas Foundation, Minas Gerais, Belo Horizonte, MG, Brazil; c Federal University of Minas Gerais, Belo Horizonte, MG, Brazil; d René Rachou Institute, Oswaldo Cruz Foundation of Minas Gerais, Belo Horizonte, MG, Brazil

**Keywords:** Leg ulcer, Sickle cell disease, Microbiome, *Staphylococcus* spp

## Abstract

**Introduction:**

Leg ulcers are a common, painful, and difficult-to-treat complication of sickle cell disease due to their prolonged healing process. The microbial composition of these wounds remains poorly understood. This study investigated the microbiota diversity in sickle cell disease leg ulcers and associations with laboratory parameters.

**Methods:**

Samples were collected from leg ulcers, intact skin adjacent to the ulcers, and intact skin from healthy controls. Microbiome profiling was performed by sequencing the V4 region of the 16S ribosomal RNA (rRNA) gene. The laboratory results of patients with sickle cell disease leg ulcers were analyzed alongside the identified taxa using correlation-based analyses.

**Results:**

Microbial diversity was significantly lower in sickle cell disease leg ulcers compared to intact skin. Additionally, leukocytes and mean corpuscular volume were statistically associated with the relative abundance of bacteria of the genera *Pseudomonas* and *Aeromonas. Staphylococcus* spp. was the predominant genus identified in leg ulcers and was negatively associated with *Pseudomonas* spp., reflecting a dysbiotic microbial profile characterized by taxa commonly associated with chronic wounds.

**Conclusions:**

The observed associations between bacterial taxa and laboratory parameters are exploratory, do not imply causality, and contribute to the characterization of the microbial ecology of sickle cell disease leg ulcers.

## Introduction

Sickle cell disease (SCD) is an inherited genetic disorder caused by a point mutation (rs334) in the *HBB* gene (Hemoglobin, beta), which encodes the beta (βs) subunit of hemoglobin [1,2]. This mutation causes erythrocyte deformation, which, together with inflammatory processes, impairs microcirculation and leads to widespread tissue damage and secondary complications, including leg ulcers [1,3].

Leg ulcers in SCD are a clinically important complication that affects 10% of the population and generally impacts the quality of life of patients [3,4]. Although the pathogenesis of SCD-related leg ulcers is recognized as multifactorial, the specific influence of the local wound microbiome on ulcer chronicity remains insufficiently characterized [3].

Next-generation sequencing (NGS) technologies have advanced our understanding of microbial diversity, and compositional shifts in the microbiome are increasingly linked to numerous chronic diseases [5,6]. In this sense, the distribution of bacterial species abundances in wound environments reveals microbial diversity, and its reduction leads to the state of dysbiosis that is associated with disease [7]. Generally, wounds have a predominant abundance of certain bacterial genera which can influence the composition of bacterial communities [[8], [9], [10]]. In SCD, exploratory studies aiming to understand the microbiota diversity in leg ulcers using NGS techniques are still scarce.

Therefore, investigating the microbial diversity of bacterial communities in leg ulcers and skin of patients with SCD is essential to characterize the microbial ecology associated with this complication. However, current evidence remains limited and largely descriptive, particularly regarding the distinction between microbial colonization and infection. Hence, the present study aimed to describe and compare the bacterial composition of leg ulcers and intact skin of patients with SCD using 16S rRNA sequencing, exploring associations with laboratory parameters.

## Material and methods

### Study design

This cross-sectional study was approved by the Research Ethics Committee. The study included samples from 72 individuals; 27 patients with SCD and leg ulcers, 23 patients with SCD without a history of leg ulcers, and 22 individuals with no history of chronic diseases. All patients with SCD had the Hb SS genotype. Controls were selected from individuals present in the Hemominas Foundation outpatient clinic on the day of leg ulcer sample collection, matched by sex and age to SCD patients. Patients, family members, and healthcare workers were excluded from the control groups.

Patient information such as treatment with hydroxyurea, use of antibiotics, history of osteomyelitis and clinical laboratory results were extracted from interviews and medical records. All participants signed an Informed Consent Form, as previously detailed [11].

### Sample collection

Intact skin samples were collected by zigzag rubbing of a defined area (4 × 4 cm) using a sterile swab immersed in standard collection fluid 1 (SCF-1) solution, as described in the Human Microbiome Project [12]. Samples from the entire ulcer bed were collected using sterile saline solution (0.9% NaCl). Intact skin samples from patients with leg ulcers were collected from the contralateral leg or the farthest point from the leg ulcer. Two swabs of intact skin samples were collected from the same defined region of each participant. All swab tips were detached using individual sterile scissors and stored in 1.5 mL tubes. The tubes were stored within two hours after collection in a freezer at −80 °C until processing.

### Sample processing and sequencing

Bacterial DNA was extracted using the FastDNA™ Spin kit (MP Biomedicals, LLC, Irvine, CA, USA), following the manufacturer's instructions. All samples were quantified using the dsDNA HS (High Sensitivity) Assay Kit on the Qubit® 3.0 fluorometer (Invitrogen™).

Polymerase chain reaction (PCR) amplification of the V4 region of the bacterial 16S rRNA gene was performed in a total volume of 25 µL containing: 12.5 µL of Q5 High-Fidelity 2X Master Mix (New England Biolabs, Ipswich, MA, USA), 0.5 µL of each primer (F515/R806, 10 µM), 3.5 µL of nuclease-free water, and 8 µL of template DNA (>5 ng of genomic DNA). The cycling conditions were 98 °C for 30 s, followed by 35 cycles at 98 °C for 10 s, 58 °C for 30 s, and 72 °C for 30 s, with a final extension at 72 °C for 2 min. The amplification products were submitted to 2% agarose gel electrophoresis and the DNA of the unconfirmed samples was concentrated by adding twice the volume of extracted DNA in absolute alcohol (100%), followed by overnight incubation at −20 °C for 12 h. The samples were centrifuged at 14,000 g for 30 min at 4 °C, and the supernatant was removed. Approximately 15 µL of solution was left in the tube, and 500 µL of 70% alcohol were added, followed by agitation and centrifugation at 14,000 g for 15 min at 4 °C. The alcohol was evaporated using a Speed ​​Vac (Thermo Fisher Scientific, Waltham, Massachusetts, USA), and the DNA pellet resuspended in 15 µL of TE (10 mM Tris–HCl, pH 8.0; 0.1 mM EDTA, Thermo Fisher Scientific) and re-amplified.

Of the 49 SCD samples collected from SCD patients with leg ulcers (G1 and G2), ten adjacent intact skin samples (G2) did not yield DNA for sequencing. The number of processed samples for the control groups (G3 and G4) was based on the number of samples in G1.

Libraries were purified using Agencourt® AMPure® XP Reagent (Beckman Coulter Life Sciences) and quantified using Qubit. The libraries were diluted and pooled together to achieve a final equimolar concentration of 45 pM. The template was prepared on the Ion Chef System (Thermo Fisher Scientific) using the Ion PGM Hi-Q View Chef kit. The Ion 318 Chip v2 was loaded and coupled to the Ion Torrent™ Personal Genome Machine (Life Technologies, Waltham, MA) for sequencing.

### Data analysis

The analysis of 16S rRNA gene sequences was performed using the QIIME2 software. DADA2 was used to filter and process sequences, retaining reads with a length of 200 bp and a quality score ≥30 to infer Amplicon Sequence Variants (ASVs) [13].

The phylogenetic tree was constructed using q2-fragment-insertion with the SATé-enabled phylogenetic placement (SEPP) method [14]. Alpha diversity metrics (Chao1, Simpson, observed ASVs, and Shannon) and beta diversity metrics (Jaccard, Bray-Curtis, and weighted and unweighted UniFrac) were calculated following subsampling to a normalized rarefaction depth of 10,000 reads per sample. Sequence quality and sampling saturation were confirmed using observed ASV rarefaction curves, and only samples demonstrating adequate coverage were retained for taxonomic classification. ASVs were classified using the Greengenes reference sequence database and their proportions were evaluated.

### Statistic analysis

Differences in diversity, taxa and clinical associations were evaluated using statistical methods. The paired Student's *t*-test or Wilcoxon signed-rank test was used for dependent groups with post hoc Bonferroni correction, whereas one-way ANOVA or the Kruskal-Wallis test was used for independent groups with post hoc False Discovery Rate (FDR) correction. Permutational analysis of variance (PERMANOVA) was used for beta diversity analysis, with the Benjamini-Hochberg Procedure for correction. Differentially abundant taxa were identified using Linear Discriminant Analysis Effect Size (LEfSe), which combines the non-parametric Kruskal–Wallis test with linear discriminant analysis (LDA) to estimate the effect size of each taxonomic feature. Features with LDA scores greater than 4.0 were considered significant. Heatmap analysis was evaluated using the Spearman’s rank correlation test. Statistical significance was set at *p* < 0.05 for all analyses.

## Results

### Patient characteristics

A total of fifty patients with SCD were included in the study, with a mean age of 39 years and 76% (38/50) received Hydroxyurea treatment. Among the patients with leg ulcers 59.3% (16/27) were women, 25.9% (7/27) had used antibiotics in the past month, and 18.5% (5/27) had a history of osteomyelitis. Additionally, leukocyte and platelet counts were significantly higher in these patients (*p* = 0.0068 and *p* = 0.0035, respectively), Table 1.

**Table 1:** Demographic and clinical characteristics of patients with sickle cell disease.

Characteristic	Patient - Hb SS			
	Leg ulcer n = 27	No leg ulcer n = 23	Total n = 50	*p*-value
**Sex** – n (%)				
Female	16 (59.3)	11 (47.8)	27 (54.0)	0.6004*
Male	11 (40.7)	12 (52.2)	23 (46.0)	
**Age** – median (range)	39.6 (±11.2)	38.4 (±12.3)	39.7 (11.2)	0.4169^a^
Use of antibiotics in the last month – n (%)	7 (25.9)	0	7 (14.0)	-
Hydroxyurea – n (%)	18 (66.7)	20 (87.0)	38 (76.0)	0.0886^⸸^
History of osteomyelitis – n (%)	5 (18.5)	1 (4.3)	6 (12.0)	0.9813^⸸^
**Laboratorial results** – median (range)				
Reticulocyte count	6.4 (1.1–12.7)	5.9 (2.6–22.1)	6.3 (1.1–22.1)	0.6549•
Leukocyte count	10,356.2 (4812–17,230)	7830.3 (4100–15,870)	9170.6 (4100–17,230)	0.0068^a^
Platelet count	401,219.2 (165,500–675,500)	293,604.3 (111,100–570,500)	350,706.1 (111,100–675,500)	0.0035^a^

*: Chi-square test; ^a^: *t*-test; ^⸸^: Fisher exact Test; •: Mann-Whitney test.

### Bacterial diversity

A total of 84 samples were sequenced, including 22 leg ulcer samples (G1), 17 adjacent intact skin samples from SCD patients with leg ulcers (G2), 23 intact skin control samples from SCD patients without a history of leg ulcers (G3), and 22 intact skin control samples from healthy individuals (G4). The sequencing generated 15,279,295 reads, with an average of 177,385 reads per sample.

Alpha diversity, assessed by the richness and evenness of taxa within the sample, revealed lower diversity in SCD leg ulcers. Significant differences were observed between leg ulcer samples and intact skin samples from SCD patients with ulcers, as well as between leg ulcers and skin samples from both SCD patients and healthy controls (Figure 1A and 1B; Supplementary Table S1 and S2), with p < 0.001. The only exception was the Simpson index (p = 0.002), which indicated lower taxonomic dominance in leg ulcers compared to the intact skin of SCD patients with ulcers. In intact skin samples, microbial diversity was similar between both SCD patient groups. However, the observed ASV count and Chao1 index were significantly lower in these groups compared to healthy control skin (p = 0.012 and p = 0.010 for G2 vs. G4, respectively; p = 0.026 and p = 0.021 for G3 vs. G4, respectively). Among control samples, taxonomic dominance, as indicated by the Simpson index, was lower in the skin of patients without a history of leg ulcers (p = 0.022) (Figure 1C and Supplementary Table S3).

**Figure 1:**
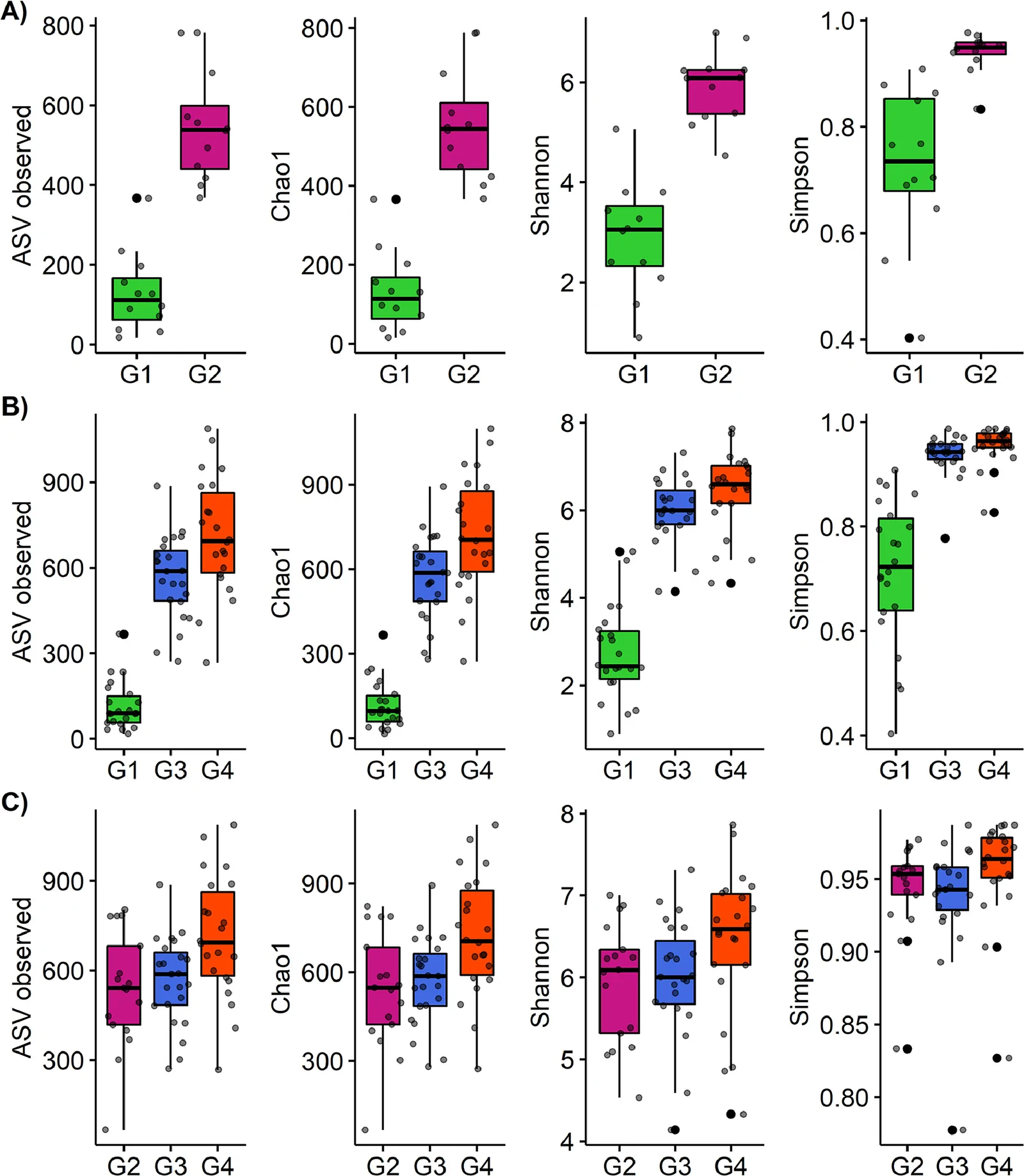
Alpha diversity and distribution across groups. A) Diversity of taxa between leg ulcers (G1: n = 12) and adjacent skin (G2: n = 12). B) Diversity of taxa among leg ulcers (G1, n = 22) and the intact skin of controls (G3, n = 23; and G4, n = 22). C) Diversity among different groups of intact skin (G2, n = 17; G3; and G4).

Analysis of beta diversity demonstrated that the microbial community structure of leg ulcer samples differed significantly (*p* < 0.005) from that of intact skin controls, with distinct sample clustering observed across all distance metrics evaluated (Supplementary Table S4). The intact skin samples from SCD patients with and without leg ulcers were also similar in terms of this diversity, except for the Jaccard index, which was close to the significance threshold (p = 0.045). Importantly, both intact skin samples from SCD patients were significantly different (p < 0.05) from the healthy control samples for all beta diversity indices.

At family levels, the most abundant bacterial taxa in leg ulcers were Staphylococcaceae (Firmicutes), Pseudomonadaceae (Proteobacteria), and Corynebacteriaceae (Actinobacteria) (Figure 2A). The genera *Staphylococcus, Pseudomonas*, Ralstonia, Corynebacterium, Arcanobacterium, Aeromonas, Stenotrophomonas, Helcococcus, *Streptococcus*, Finegoldia, Pelomonas, Anaerococcus and Sphingomonas were more abundant in leg ulcers (Figure 2B). Ralstonia, Corynebacterium and *Staphylococcus* were the three most abundant taxa in the intact skin groups in this order.

**Figure 2:**
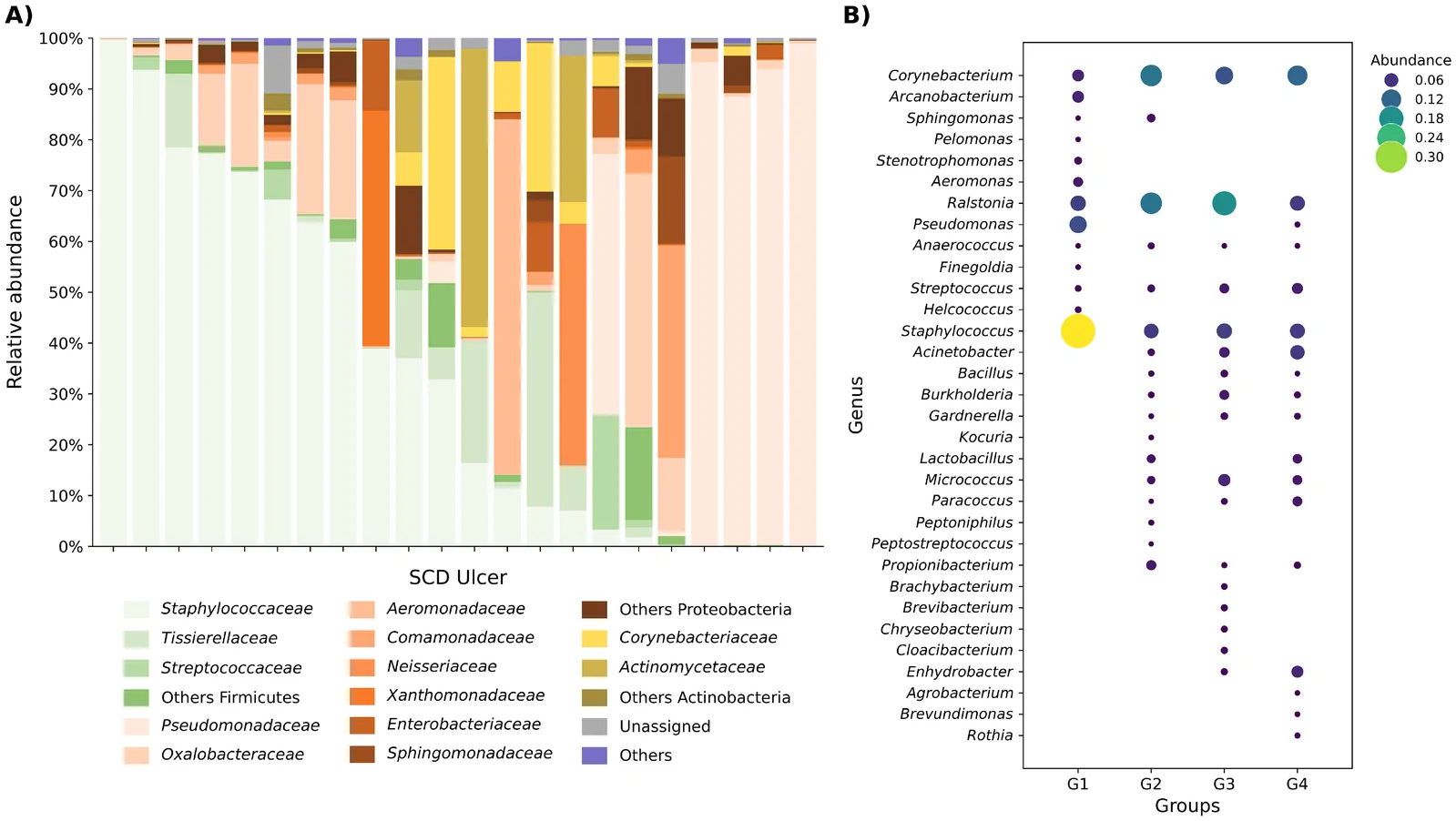
Bacterial composition of leg ulcer and intact skin. A) Bar plot of mean relative abundance >1% of dominant families in leg ulcer. Others refers to bacterial taxa below 1%. B) Distribution taxonomy of abundant genera among groups.

The overrepresented genera were assessed for significance, considering a median relative abundance greater than 1%. The taxon *Staphylococcus* was the most abundant genus in leg ulcers, but no significant differences were observed when compared with the control groups (Supplementary Table S6). *Corynebacterium* and *Streptococcus* genus were significantly less abundant in leg ulcer samples, while *Ralstonia* was lower only in comparison with intact control skin from SCD patients (p = 0.002). The intact skin of patients with leg ulcers showed no differences when compared to controls (Supplementary Table S7).

In the evaluation of the differential abundance of total taxa, the genus *Aeromonas* was associated with leg ulcers, while the relevant genera for the control skin samples of SCD patients were *Ralstonia, Micrococcus*, and *Burkholderia*. In the healthy control group, the genera associated with higher relative abundance were *Corynebacterium, Acinetobacter, Enhydrobacter, Paracoccus, Lactobacillus*, and *Streptococcus* (Figure 3C). Furthermore, the genera most represented in SCD leg ulcers were evaluated to explore statistical associations with laboratory parameters. Laboratory results showed statistically significant correlations with overrepresented taxa, with a positive association for leukocytes: *Pseudomonas* (p = 0.014), Hemoglobin F: *Streptococcus* (p = 0.024), and a negative association for mean corpuscular volume (MCV): *Aeromonas* (p = 0.040) (Figure 3B and Supplementary Table S7). *Arcanobacterium* and *Helcococcus* were strongly correlated in SCD leg ulcers (p < 0.001), while the genera *Staphylococcus* and *Pseudomonas* were negatively associated (p = 0.002), (Supplementary Table S8).

**Figure 3:**
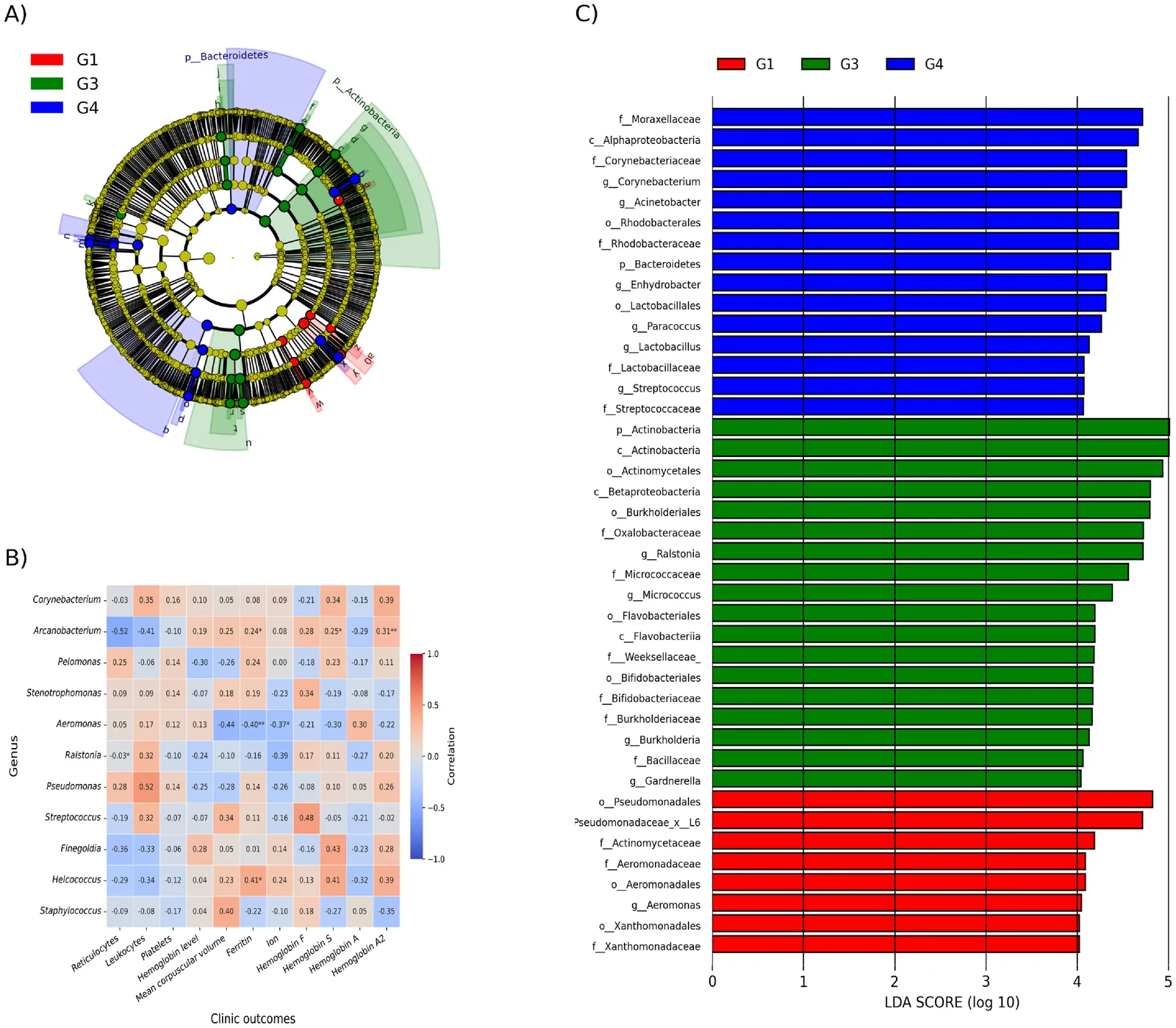
Associations with leg ulcer and intact skin control samples. A) Cladogram of taxon distributions by groups. B) Correlation heatmap of laboratory parameters and genus of leg ulcers. C) Distribution taxonomy of differential abundance among groups of leg ulcer and controls.

## Discussion

This study provides a descriptive characterization of the microbial composition of leg ulcers in patients with SCD, compared with intact skin from SCD patients and healthy controls. The reduced alpha diversity and distinct beta diversity clustering observed in leg ulcer samples are consistent with features commonly reported in chronic wounds, rather than representing a disease-specific microbial signature unique to SCD.

In this study, lower alpha diversity and distinct clustering of beta diversity were observed in SCD leg ulcer samples compared to intact skin samples. These findings likely reflect the chronic wound environment, characterized by local inflammation, hypoxia, tissue damage, and altered nutrient availability, rather than a direct causal effect of systemic SCD-related factors alone [15,16]. However, the intact skin of patients with leg ulcers and the control group with SCD showed similarities in their clusters. These results suggest that the richness of the skin microbiome in SCD exhibits distinct disease characteristics compared to healthy skin, independent of the leg ulcer complication.

These findings are consistent with a previous study that analyzed the microbiota of leg ulcers in SCD patients [8], which reported an overrepresentation of *Staphylococcus aureus*, similar to observations in diabetic foot ulcers and atopic dermatitis [17,18]. The species *Staphylococcus aureus* is an opportunistic commensal pathogen of the human microbiota, associated with several chronic health conditions, and is commonly found in the nasopharyngeal region of individuals with SCD [[19], [20], [21]]. *Staphylococcus aureus* has been associated with the progression of skin lesions in cutaneous squamous cell carcinoma and, along with *Pseudomonas aeruginosa*, has been linked to delayed healing and persistence of foot ulcers in diabetics [[22], [23], [24], [25]]. Here, the abundance of *Pseudomonas* was also overrepresented in SCD leg ulcers, consistent with reports of its presence in chronic wounds due to its role in biofilm formation [15,26].

Differential abundance analysis of intact skin samples indicated that the genera *Corynebacterium, Ralstonia, Streptococcus, Micrococcus, Burkholderia, Acinetobacter, Enhydrobacter, Paracoccus*, and *Lactobacillus* were significantly more abundant than in SCD leg ulcers. Meanwhile, LEfSe analysis revealed the opportunistic pathogen *Aeromonas* to be significantly associated with SCD leg ulcers. Additionally, other important taxa, such as *Pseudomonadaceae, Aeromonadaceae, Xanthomonadaceae* and *Actinomycetaceae*, were also significantly associated with SCD leg ulcers. These taxa have been widely reported in polymicrobial chronic wound environments and are not exclusive to SCD-related ulcers [15,24,27].

Correlation analyses identified statistical associations between selected laboratory parameters and bacterial taxa; however, these findings should be interpreted with caution. For example, leukocyte counts are non-specific markers influenced by infection, inflammation, stress, and other systemic conditions. Thus, the observed association between leukocyte levels and *Pseudomonas* abundance does not establish a causal or mechanistic relationship. Similarly, associations involving hemoglobin fractions, mean corpuscular volume, and specific taxa may reflect indirect or confounded relationships rather than direct biological effects [1,2,16,17].

Importantly, bacterial detection in this study was based on 16S rRNA gene sequencing, which identifies bacterial DNA but does not distinguish between colonization and active infection. The presence of bacterial DNA alone does not indicate high microbial burden or clinically relevant infection capable of impairing wound healing. Furthermore, clinical signs of infection were not systematically assessed at the time of sampling [12,[14], [15], [16],^25^,^27^].

Taxa were correlated with each other, but in particular *Staphylococcus* and *Pseudomonas* were negatively associated in SCD leg ulcers. This finding is consistent with competitive interactions previously described in chronic wound microbiomes and likely reflects ecological dynamics rather than pathogen-specific dominance [25,28].

This study has several limitations. Its cross-sectional design precludes causal inference, and wound duration, antibiotic exposure, local wound care practices, vascular status, and comorbidities such as diabetes or iron overload were not fully controlled. Additionally, 16S rRNA sequencing does not allow species-level identification or functional assessment of microbial activity, virulence, or antibiotic resistance. The sample size also limits subgroup analyses and generalizability.

## Conclusion

This study characterizes the microbial ecology of SCD leg ulcers as a dysbiotic, chronic wound-associated community. The findings should be interpreted as exploratory and hypothesis-generating, providing a foundation for future longitudinal and mechanistic investigations integrating clinical infection data, wound chronicity, and functional microbiome analyses.

## Data availability

The data that support the findings of this study are available from the corresponding author upon reasonable request.

## Conflicts of interest

The authors declares no conflicts of interest.

## Appendix Supplementary materials

Image, application 1

## Footnotes

Supplementary material associated with this article can be found in the online version at doi:10.1016/j.htct.2026.108550.
